# A Systematic Review of Peer-Reviewed Studies on Preventing Sport-Related Concussion (SRC) in Adult Football (Soccer): Mapping Sparce Evidence of Rule Changes and Head-Neck Training

**DOI:** 10.3390/healthcare14091200

**Published:** 2026-04-29

**Authors:** Sonya Moore, Teale Vella, Jessica Norton, Kai Lin Foong, Mitchell Barbara, Chris Musgrave, Kuan-Yin Lin, Jennifer R. A. Jones

**Affiliations:** 1 Faculty of Medicine, Dentistry & Health Sciences, The University of Melbourne, Parkville 3052, Australia; tealevellaphysio@gmail.com (T.V.); jessica.norton@physiologic.com.au (J.N.); kailin.foong@student.unimelb.edu.au (K.L.F.); mitch@catalystsportsclinic.com.au (M.B.); c.musgrave@unimelb.edu.au (C.M.); jennifer.jones@unimelb.edu.au (J.R.A.J.); 2 School and Graduate Institute of Physical Therapy, College of Medicine, National Taiwan University, Taipei City 100025, Taiwan; kuanyinl27173@ntu.edu.tw

**Keywords:** football, soccer, concussion, traumatic brain injury, sport-related concussion, injury prevention

## Abstract

**Highlights:**

**What are the main findings?**

- Current evidence is scarce, heterogeneous, and in infancy stages and therefore insufficient to determine any specific interventions and/or protocol features which are effective in preventing SRC.
- Peer reviewed evidence is limited to non-randomized studies investigating rule change and head-neck training and interventions.
- Rule changes to mitigate head impacts and head-neck training are grounded in clinically sensible speculative mechanism of effect, but have no proven impacts.

**What are the implications of the main findings?**

- Current evidence is insufficient to inform definitive practice recommendations for preventing SRC in soccer.
- Soccer-specific primary research is needed to substantiate any significant effects of interventions including rule changes, head-neck training, and broader interventions.
- Despite ambiguous benefits, clinically sensible rule changes to mitigate head impacts and head-neck training have been feasibly implemented in competitive soccer leagues, team training and non-experimental research environments.

**Abstract:**

**Background/Objectives**: Acute health impacts and longer-term sequelae of sport-related concussion (SRC) are recognized concerns in football (soccer), warranting investigation of interventions to reduce the incidence. The purpose of this study was to identify, synthesize and evaluate interventions used in preventing sport-related concussion (SRC) in adult soccer players. **Methods**: Five databases (MEDLINE, CINAHL, Embase, SPORTDiscus, PsycINFO) were searched on 6 September 2024 and updated on 17 December 2025 for concussion prevention intervention studies involving adult footballers. Study quality was assessed with the Modified Downs and Black Checklist. A narrative synthesis of all included studies followed Synthesis Without Meta-Analysis (SWiM) guidelines. **Results**: From 3463 records, five studies met inclusion criteria: three reported rule changes and two reported head-neck training interventions. The low volume of studies discovered were non-randomized and rated fair or poor on quality assessment. Whilst these interventions were grounded in sound and well-reasoned mechanisms to mitigate SRC risks, none reported statistically significant directional effects. This, combined with high heterogeneity, prevented data pooling and no firm conclusions could be drawn about the effectiveness of any intervention. **Conclusions**: Sparce, preliminary, heterogeneous evidence represents research to reduce SRC in adult soccer players, and this is limited to investigating rule changes and head-neck training and interventions. A larger volume of primary research is needed to determine meaningful practice recommendations of these and other conceivable interventions.

## 1. Introduction

Sport-related concussion (SRC) warrants global sports-medicine-related advocacy [1] and public health awareness, with annual estimates of 3.8 million cases in America [2] and 100,000 in Australia [3]. Up to 10% of athletes experience persistent neurocognitive symptoms including headaches, sleep disturbances, and balance problems lasting more than two weeks [4] with prolonged symptoms costing up to four times more than shorter-duration injuries [5]. Retired athletes with repetitive SRC show poorer performance across multiple cognitive domains compared to controls [6]. Compulsory short-term participation restrictions [7] and an emerging understanding of long-term SRC sequelae, incentivize government and sporting bodies to prioritize primary and secondary prevention strategies [8]. In practical healthcare terms, primary prevention refers to actions which mitigate the likelihood of concussion occurrence in the first instance, e.g., protocols, education and policy changes; and secondary prevention refers to actions which mitigate further injury subsequent to the first instance. Specific to soccer the collaborative FIFA and World Health Organization (WHO) “suspect and protect” campaign requires athletes with suspected concussion to be removed from participation pending further assessment [9]. Targeted prevention could reduce healthcare burden, limit injury impact, and support safe lifelong participation in sport [1].

This review adopts the Concussion in Sport Group (CISG) definition of sport-related concussion (SRC) synonymous with a mild traumatic brain injury (mTBI) from direct impact to the head, neck, or body, transmitting impulsive forces to the brain during sports or exercise [1]. These forces trigger metabolic changes, leading to altered cerebral blood flow, inflammation, and in severe cases, axonal injury [3]. Clinical signs may include loss of consciousness, altered mental status, amnesia, confusion, disorientation, headache, nausea, dizziness, balance deficits, light/noise sensitivity, cognitive impairments including mental fog, concentration, memory issues, or emotional changes such as lability or irritability [10]. Symptoms can appear immediately or be delayed by minutes to hours and may persist for days or longer [1,10]. Together, the accumulation of evidence on SRC prevalence, clinical features and burden highlights the complexity of SRC and reinforces the urgent need for effective prevention strategies.

The football code of soccer involves play using the head to direct the ball (heading) and competitive aerial challenges, which raises the risk of head injuries, including SRC [11]. Soccer’s international appeal is estimated in excess of 240 million players worldwide from grass-roots participation to elite professional ranks [12]. Rates of SRC have risen as definition, recognition and reporting have evolved with head injuries currently accounting for 4–22% of all soccer injuries [13]. Head-to-head contact during collisions and aerial challenges are the most common SRC mechanism in adult soccer [14,15]. Consequently, examinations of soccer-specific interventions and outcome data are imperative to support SRC prevention and reduction initiatives spanning grassroots participation, professional performance and long-term health of veteran players. Furthermore, structural, functional and developmental differences between child, adolescent and adult brains have informed development of age-specific SRC guidance, alongside different sport-participation and/or competition agendas [7]. This requires that evidence be specifically aligned to meet both sports-specific needs of age-specific populations.

Targeted SR of available evidence is essential to inform the design and direction of future primary research to inform clinical practice. Recent systematic reviews have reported that the standardized neuromuscular FIFA 11+ protocol can reduce head-neck injuries in soccer players [16]; low quality evidence supporting headgear for prevention of SRC in adolescent female soccer players has also been reported [17]. FIFA Medical-governed projects are underway to systematically search and summarize all existing research relating to football heading, to create an open-source, online Heading in Football Research Database [18]. Specific to heading the ball outcome measures, this substantially differs from our review, which focusses on all/any head-impact mechanisms leading to diagnosed SRC. Although repeated heading may pose long-term brain health risks [19] it is not a common mechanism of acutely diagnosed SRC [20].

Despite these valuable adjacent inquiries into precise aspects of SRC in soccer, no reviews have systematically searched the evidence to address the more open overarching question of what interventions have been researched, and what interventions are effective for SRC prevention in adult soccer players? Specific and nuanced investigation of various, different and sub-entities of SRC in soccer is highly valuable, underpinned by the shared value system of informing player wellbeing. However, answering this overarching question is fundamental to mitigate a piecemeal-scattergun approach to meeting the urgent need for evidence informed soccer-specific practice recommendations and identifying strategic directions for future randomized research designs. No planned or current reviews addressing this question were registered on PROSPERO or the Cochrane Database of Systematic Reviews.

This systematic review therefore aims to examine the effectiveness of all/any interventions in preventing SRC in adult soccer players. Objectives were to identify interventions used in the prevention of SRC in adult soccer players; and to synthesize and evaluate the effectiveness of these interventions. The impact of this review’s findings can be used to inform concussion prevention research and practice, improve health outcomes for adult soccer players and reduce the associated healthcare burden. Additionally, this review will provide evidence to inform athletes, coaches and support staff on effective strategies towards reducing the risks of SRC.

## 2. Materials and Methods

This systematic review was conducted and reported in accordance with PRISMA 2020 guidelines [21], with the checklist provided in Supplementary File SA. Prior to any data extraction, the review protocol was prospectively registered with PROSPERO (CRD420251063951) [22] and no changes were made after the protocol was registered.

A comprehensive search strategy of five databases (Ovid MEDLINE, EBSCOhost CINAHL, Ovid Embase, EBSCO SPORTDiscus and EBSCO APA PsycINFO) was performed on 6 September 2024 and updated on 17 December 2025. Although searching gray literature sources could have reduced publication bias, limiting the search to peer reviewed databases preserved standardized methods, reporting and quality [23]. Search terms were based on three main concepts: football (soccer), concussion and prevention strategies. MeSH terms for brain concussion, along with keywords, intervention terms, and truncated terminology, were used to maximize the retrieval of relevant studies. The full search strategy designed in consultation with a health information librarian is presented in Supplementary File SB. The sensitivity of this search strategy is represented by the following full MEDLINE search string:

MEDLINE Search string:

1. Brain Concussion/
2. concuss*.mp.
3. mild traumatic brain injur*.mp.
4. mtbi.mp.
5. 1 or 2 or 3 or 4
6. Football.mp.
7. soccer.mp.
8. 6 or 7
9. Prevent*.mp.
10. (reduce or reduces or reduced or reduction* or reducing).mp.
11. Equipment.mp.
12. (Mouthguard* or mouth guard* or mouthpiece* or mouth piece*).mp.
13. (Helmet* or head gear* or headgear*).mp.
14. ((resistance or proprioception) adj2 (train* or exercis*)).mp.
15. Neuromuscular training*.mp.
16. (risk or risks).mp.
17. ((Policy or policies or rule* or training or game*) adj2 (chang* or amend* or modif* or regulation* or legislation*)).mp.
18. ((muscle* or neck or cervical or core) adj2 (strength* or training)).mp
19. (rest or rests or resting or rested).mp.
20. (Nutrition* or diet).mp.
21. Vitamin*.mp.
22. supplement*.mp.
23. Education* adj2 (program* or intervention*)).mp.
24. 9 or 10 or 11 or 12 or 13 or 14 or 15 or 16 or 17 or 18 or 19 or 20 or 21 or 22 or 23
25. 5 and 8 and 24
26. limit 25 to (english language and yr = “2001 -Current”)

Publications from 2001 onward were eligible, aligning with the first International Symposium on Concussion in Sport, where the contemporary definition of concussion was established [24]. This is consistent with existing SRC research [25].

The study selection criteria are presented in Table 1. Studies were included if they evaluated a SRC preventative intervention in adult footballers aged eighteen years or over, of any reported sex or gender and at any performance level (recreational, semi-elite, professional). Interventions in eligible studies could be compared to “usual care” in the context of soccer participation and SRC risk mitigation, which was defined to resemble care and injury intervention activities normally provided as standard practice or a control group which did not receive a novel intervention [26]. This catered to inclusion of study designs including randomized controlled trials (RCTs), quasi-randomized controlled trials, prospective and retrospective cohort studies. Anticipating the shifting landscape of RCT research design primacy in fast-moving clinical investigations [27], including pre–post designs without a concurrent control group was important to capture emerging interventions yet to be evaluated through randomized study designs and to enable potential comparisons between intervention, no intervention and/or standard practice. While high-quality RCTs provide the strongest evidence on intervention efficacy, including non-randomized studies in systematic reviews can help assess how the overall evidence applies to routine clinical practice and fill gaps in the RCT evidence base [28,29]. The articles included must have reported a SRC incidence-related outcome, including medically diagnosed SRC, athlete-reported SRC, or incidence rates. Only peer reviewed, full text, human research articles published in English were included.

Following the search, all identified papers were uploaded to Covidence [31] and duplicates removed. Screening titles and abstracts, and reviewing full texts were conducted independently and in duplicate by three reviewers (JN, TV, SM). Systematic reviews which met all population and outcome inclusion criteria were progressed through to the full text screening stage, citation lists were hand searched, and were then excluded based on study design. Citation lists of included studies were hand-searched.

Data extraction was performed by two independent reviewers using a customized and pre-piloted Covidence data collection tool. All authors contributed to the data extraction process by inputting agreed data items into an Excel spreadsheet. Data items extracted included (1) studies characteristics (e.g., study title, authors, publication year, country, study design); (2) population characteristics (e.g., number of athletes, age, sex, performance level); (3) prevention strategy (e.g., intervention type, season/s delivered); (4) pre-intervention group (e.g., type, season/s); and (5) the results and outcomes (e.g., SRC incidence per 1000 h or number of incidents per time period).

Methodological diversity, limited evidence and data characteristics did not meet our a priori registered protocol criteria to undertake meta-analysis [22]. Given the low volume of studies with substantially different intervention characteristics, a narrative synthesis was performed for all included studies following the Synthesis Without Meta-Analysis (SWiM) guidelines [32] to offer interpretation, critique and deeper understanding [33]. The full SWiM Checklist is presented in Supplementary File SC.

Quality assessment was performed independently and duplicated by two authors using the Modified Downs and Black Checklist. This was an a priori single tool to score quality, internal and external validity of both randomized and non-randomized study designs [34] which has been applied in other concussion research [35]. A third reviewer was available if consensus was not reached. Downs and Black score ranges were given corresponding to quality levels as previously reported: ≤14 was considered poor, 15–19 fair, 20–25 good and >26 excellent. The full Quality Assessment Modified Dows and Black Checklist is presented in Supplementary File SD.

## 3. Results

### 3.1. Study Selection

**Figure 1:**
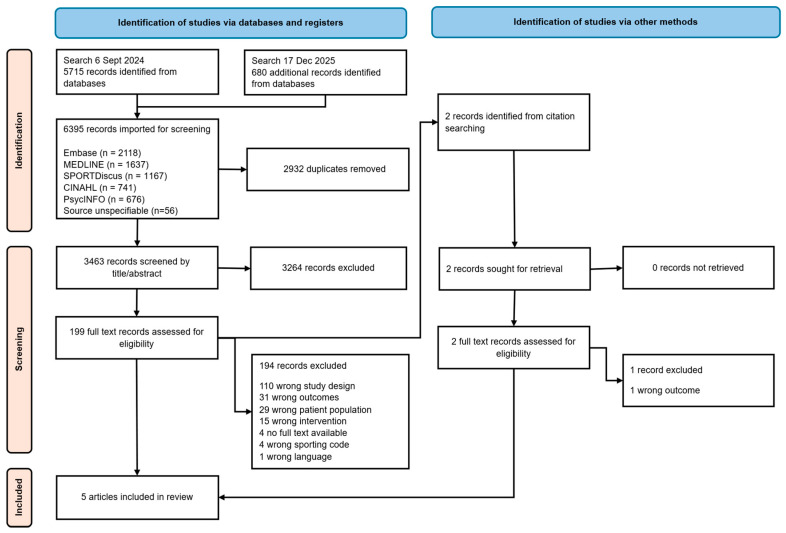
Study flow diagram according to Preferred Reporting Items for Systematic Review and Meta-Analyses (PRISMA) 2020 Flow Diagram.

A total of 6395 records were retrieved from the database search and uploaded to Covidence. Figure 1 illustrates the PRISMA flow diagram of the search process. After the removal of duplicates and non-eligible records, 3463 records were independently title/abstract screened by two authors. Two records were retrieved from hand-searching citation lists of relevant systematic reviews. 199 records were assessed for eligibility by full text review. There were no selection disagreements requiring consensus resolution from a third author. Records that did not meet the inclusion criteria were excluded for reasons including incorrect study design (*n* = 110), incorrect patient population (*n* = 29), incorrect outcomes (*n* = 31) and no SRC prevention intervention (*n* = 15). There were four exclusion reason conflicts which were resolved by third reviewer consensus. A total of five articles (*n* = 5) were included in this review.

### 3.2. Study Characteristics

The characteristics of included studies are presented in Table 2. Three studies evaluated effects of rule changes (*n* = 3) in professional leagues in Germany [36,37] and Norway [38]. Two studies evaluated the effects of head-neck training interventions (*n* = 2) [4,39] in collegiate level soccer in North America [4,39]. Study designs included two prospective cohort studies [4,38], one retrospective cohort study [36], one combined retrospective and prospective cohort study [37] and one single arm interventional study [39].

All participants were in collegiate [4,39] or professional adult soccer leagues [36,37,38,40] and either reported as [4,38,39], or therefore assumed as [36,37], adults over the age of 18. The total number of individual participants could not be calculated since some studies reported participation according to the number of league teams [36,37,38].

Sex and gender data were extracted as they were reported, acknowledging this finding as a binary framework of reference. Rule change studies contained only male participants in male soccer leagues [36,37,38]. Head-neck training intervention studies reported both male and female participants (53% male, 47% female) [4,39].

All studies reported intervention outcomes as different SRC-related metrics. This included either SRC number per season (*n*SRC) or concussion incidence rate (IR-SRC) per 1000 h. Four studies [4,36,37,38] further differentiated relative risk exposure metrics of match/game hours, and one [39] also included training hours. Both head-neck training intervention studies presented concussion data combined with either (a) traumatic lower extremity musculoskeletal injuries [39] or (b) concussion mechanisms from different sports including soccer [4]. This data could not be extracted separately. Only two studies reported participant attrition, which were due to withdrawal from sport, noncompliance to protocol, or unspecific reasons [4,39].

### 3.3. Summary of Findings

Table 3 presents a summary of study outcomes and effect estimates for the five included studies grouped by preventative intervention type (i.e., rule changes or head-neck training interventions). All available results for individual studies are reported, acknowledging specific inconsistencies in confidence interval reporting and team versus individual player participant numbers (*n*-value).

#### 3.3.1. Rule Changes

Two studies [36,37] assessed the impact of a 2006/07 International Football Association Board (IFAB) rule change penalizing deliberate elbow-to-head contact with a red card. After the rule change between 2007/08 and 2012/13 seasons, *n*SRC dropped from 50 to 35 and IR-SRC decreased from 0.67 (95% CI 0.51–0.88) to 0.48 (95% CI 0.34–0.66) per 1000 playing exposure hours, representing a 29% reduction [36]. Since this did not meet the criteria of statistical significance, true effects could not be differentiated from natural variation. A time-trend study followed the rolling season-on-season pattern following this same rule change over 11 seasons from 2006/07 to 2016/17, reporting reduced IR-SRC from 0.67 (95% CI 0.51–0.88) to 0.53 (95% CI 0.42–0.67) per 1000 playing exposure hours [37].

One study, Bjørneboe et al. [38], implemented a stricter enforcement of the red card rule change, issuing automatic red cards for late or two-footed tackles, tackles with excessive force, and intentional high elbows in heading duels. Across a pool of 12 teams in 2010, and 14 teams in 2011, *n*SRC increased from three (2010) to four (2011) pre-to-post intervention. IR-SRC per 1000 h were not reported due to a small sample size. As only one season of data was presented with a small sample, true effects of this rule change could not be differentiated from incident reporting methods or natural variation.

#### 3.3.2. Head-Neck Training Interventions

Two studies [4,39] examined head-neck training interventions to reduce the SRC risk in adult collegiate level soccer. Both reported limited data and no statistically significant effect on SRC risk reduction. No adverse events or effects were reported.

Klein et al. [4] implemented cervical stretching and progressive resistance exercises (12 week program, 45 min, 2 × weekly). Conflicting directional effects were reported, including reduced concussion incidence from 11.54 to 6.67 per athletic event in conglomerate data from all participants in men’s soccer, women’s soccer, lacrosse, hockey and wrestling athletes; and increased *n*SRC from six in 2020/21 to seven in 2021/22. Confidence intervals were not reported and there were no statistically significant effects.

Reneker et al. [39] implemented a multi-modal exercise intervention including cervical neuromotor control, strength, vestibular, visual, oculomotor training, and postural and balance exercises (4 week program, 10 min, 3 × weekly). Confidence intervals and *n*SRC were not reported. The described reduction in IR-SRC in men and women soccer participants from 11.8 per athlete exposures in 2017 to 8.94 in 2018, was not a statistically significant effect.

#### 3.3.3. Quality Assessment

The quality assessment for included studies according to the Modified Downs and Black Checklist [34] is reported in Table 2 with the full checklist for each included study in Supplementary File SD. Each study was scored against a maximum of 28 points with a higher score indicative of higher methodological quality, according to the scale presented by O’Connor et al. [41]. Both head-neck training intervention studies [4,39] were rated as fair (Scores: 15, 18), two rule change studies [36,38] were fair (Scores: 17, 14) and one rule change study [37] was poor (Score: 13).

## 4. Discussion

### 4.1. Results Synthesis in the Context of the Available Field of Evidence

This systematic review identified five studies reporting specific rule changes and head-neck training interventions intended to reduce SRC in soccer. There was an overall low volume of studies, significant statistical heterogeneity, variation in study populations and study design limitations (all were pre- post-interventional cohort studies lacking randomization). Indeed, this evidence scarcity validates our decision to include both randomized and non-randomized designs, in anticipation of RCT infancy/paucity in a medical landscape prioritizing rapid-implement clinical interventions with real-world impact observations [27].

**Figure 2:**
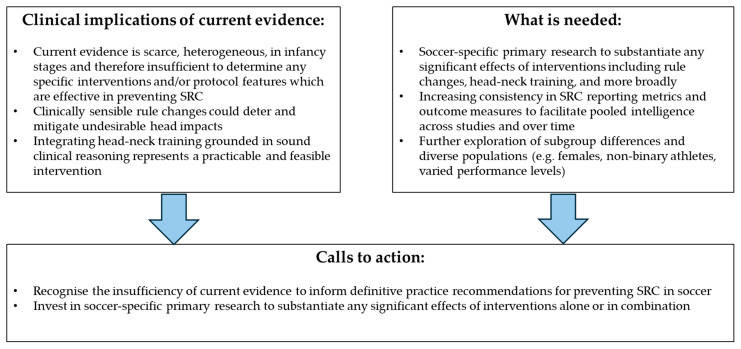
Clinical implications, needs and calls to action for SRC prevention in soccer. This figure captures a summary of itemized clinical implications, needs and calls to action which synthesize our review findings with recommendations from the broader field of SRC evidence. Abbreviations: SRC = sports-related concussion.

We therefore discerningly position discussion of our findings as cautious mapping of the preliminary literature rather than a synthesis capable of supporting intervention-oriented conclusions. We emphasize the need for vast primary research, meanwhile offering some guidance towards practical implications for SRC prevention in the infancy stage of supporting evidence. Figure 2 offers a summary of itemized clinically sensible opportunities, needs and calls to action which synthesize our review findings fittingly with the broader field of SRC evidence.

Given the identified risk and emerging longitudinal brain health data [42], we propose that theoretical models and clinically sensible albeit speculative mechanisms are valid grounds for implementing risk mitigating interventions in the current climate of protecting soccer players from head impact sequelae. This concurs with other systematic reviews which, despite a small number if included primary studies on distinctive SRC topics, also advocate for implementing prevention strategies to meaningfully reduce SRC risks [16,17,43]. Specific interventions, proposed clinically reasoned mechanisms of effect and association with SRC incidence are therefore worthy of discerning scrutiny and future development.

Our review identified two domains of SRC prevention interventions in adult soccer players. Firstly, rule-changes, which could be internationally defined as Laws of the Game at every level, or organizational level regulations [44]. Secondly, head-neck training interventions, which are similar in principle to other neuromuscular training strategies such as FIFA11+, which are well established practices to mitigate soccer injuries generally [45,46] and head-neck injuries specifically [16]. Both rule changes to reduce head impacts and head-neck training interventions are focus areas for concussion prevention in the SRC Consensus Statement [1]. These therefore represent initiatives informed by the best available evidence and can be confidently explored and integrated in a soccer-specific context.

Rule-based interventions to mitigate deliberate and accidental body and head contact are clinically sensible, and research to support soccer-specific initiatives would parallel rule changes which have been successful in reducing SRC in other sports [24,47]. In 2015, the United States Soccer Federation banned heading the ball in youth football to reduce head contacts, but this counterintuitively resulted in increased reported concussion injuries compared to other injuries [48]. Notwithstanding limited evidence relating to varied attitudes, knowledge and concussion-responsive practices [49], this raises consideration that the type of head contact impacting the head may be significant in SRC prevention in soccer. This is supported by Beaudouin et al. [36] who identified head-to-head (38%) and elbow-to-head (16%) as the most common mechanism of injury, as opposed to head-to-ball. Rule changes limiting deliberate head contact would therefore seem a common-sense intervention to reduce SRC.

Our review discovered two variations in a rule change, including (a) penalizing deliberate elbow-to-head contact with a red card [36,37], and (b) penalizing other risky body-contact mechanisms such as late or two-footed tackles, tackles with excessive force, and intentional high elbows in heading duels with a red card [38]. Whilst practically sensible, the study quality and data criteria were insufficient to conclude any effect on SRC prevention. Furthermore, constructing the deterrent enforcement criteria are both independent and inseparable from crafting a discerning rule change. Red card sanctions were introduced as the only proposed consequence and other penalty options according to impact severity circumstances may be appropriate and effective. Penalizing head contact may have limited impact on overall injury rates where skill errors and forceful head contact are unintentional, with coach–player education likely necessary in the transition towards consistently and skillfully executing desired technique changes [50].

Beyond punitive consequences, respecting the intention of any rule change would be important in promoting stakeholder and player commitment towards authentic behavior change [51]. The existing literature supports the implementation of rule-based interventions on concussion prevention in other sports, with the caveat of scrutinizing rather than presuming what could be most effective [52]. Further consultation is needed to craft rules specifically to mitigate undesirable head-impacts, accompanied by evaluation of the rule’s outcomes and influence on the game [44].

Future primary research to discern any effective rule-based interventions to mitigate SRC risks should also consider preserving characteristic soccer game-play skills. Reducing head-to-head and player-player contact could be well-envisioned targets for future SRC prevention strategies and may also be considered to cultivate skillful flow of the modern game. Antagonistically, there is growing evidence linking neurophysiological effects with repetitive heading [19], i.e., ball-to-head contact. Heading analytical models demonstrate significant and variable forces [53] and potential long term impacts of cumulative exposure to repetitive, sub concussive head-impacts (RSCIs) [20]. RSCIs have similar inciting mechanisms but without the acute clinical symptom presentation of diagnosable SRC [54]. Indeed, the term of reference RSCI requires judicious interpretation relative to any inciting force characteristics and diagnosable SRC symptoms provoked [55]. Notably absent in our review were any studies on any heading-related SRC prevention interventions, granted our selection criteria of diagnosed SRC excluded studies investigating RSCIs from consideration. Heading is a fundamental part of soccer, making modifications contentious. Prevention interventions relating to heading and the nuanced consideration of the medical diagnosable SRC and RSCI spectrum and relative risk exposure therefore warrants further high-quality research.

Notwithstanding intentions to mitigate and reduce SRC risks, player contests, body contacts and headers are integral aspects of today’s modern soccer game. Research into interventions that could reduce the forces experienced during these inevitable contacts—such as head-neck training interventions—may also offer clinically sensible and viable alternatives. Although head-neck training intervention research in our review was sparce and insufficient to determine clinical recommendations, they could be considered a valid clinically reasoned adjunct within the pragmatic paradigm of aggregated injury mitigation efforts. Mounting evidence postulates neck-training interventions could reduce head-impact forces in soccer [56] and can reduce SRC incidence in other football codes [57,58,59]. Acknowledging uncertain benefits, Peek et al. [56] outline an example of how a basic soccer-specific head-neck training program might be integrated into regular team trainings as part of the well-established FIFA-11 musculoskeletal injury prevention program. These practices are grounded in neuromuscular training principles, exercise prescription science and have no reported or speculative adverse effects. Future primary research could explore any protocol features with superior effects to another, and compare any benefits of soccer-specific programs.

High quality systematic reviews have examined the effects of policy modification, protective equipment and any unintended confounding effects of SRC preventative measures in sports other than soccer [44,47]. This review did not discover studies on protective equipment in soccer and further research is needed to substantiate any effectiveness or value-added protective equipment in soccer.

Value-based healthcare seeks to transform volume-based models to efficient outcome-optimizing interventions [60], which could support targeted and/or multi-modal interventions for SRC prevention according to contextualized risks. Like our review findings, other evidence highlights the validity of multiple, interlinking and aggregated meaningful considerations, with insufficient data to definitively conclude which interventions, if any, could have superior benefits. This concurs with established perspectives that SRC risks and management are multifactorial [1], and prevention strategies could logically align with individual player risk-profiles and include assessment-aligned interventions [25]. This would need to be informed by further primary research substantiating any effects of rule changes, head-neck training interventions and other emerging strategies. These opportunities could be expanded with sophisticated modeling, broader context consultation and high-quality primary research seeking value-assure interventions and optimized impacts.

### 4.2. Limitations

Pursuing a timely and clinically meaningful inquiry encountered significant limitations requiring acknowledgment for purposes of methodological transparency, findings interpretation and declaring unanswered questions. While our review sought to include all RCT and related study designs to capture the broadest range of high-quality emerging research in this field, common but limiting restrictions included omission of gray literature and non-English language sources which could have introduced publication and language bias to our findings. Given all discovered studies were non-RCTs with poor-fair quality assessment and heterogeneous characteristics, contacting individual study authors to request additional data was not undertaken. However, this could have added value to our review where soccer-specific and SRC data were diluted within multi-sport study and musculoskeletal injury contexts. Insufficient data was available to perform a subgroup analysis according to performance level; or reported sex or gender differences. Studies reported outcomes from male participants only, or inseparable male/female participants which prevented any discernment of sex, gender or non-binary differences. This is important for future research since sex and gender demographics can be poorly captured despite specific differences in concussion causes and outcomes [61]. We included studies reporting diagnosed SRC, rendering consideration of multiple career SRCs or RSCIs beyond the scope of this review. These differentiations would be relevant when aligning new interventions with proposed mechanisms of effect. The validity of our findings are limited by available studies, reported data and the intended scope of our review.

### 4.3. Summary of Implications for Practice, Policy, and Future Research

Our review highlights the low volume of data and primary research of interventions available to inform SRC prevention specifically in soccer. Based on the synthesis of currently available evidence, we recommend further high-quality primary research to discern specific effects of interventions in the two identifiable domains of rule changes; and head-neck training; and more broadly. In a paradigm of judiciously balancing strategic and immediate opportunities to protect player wellbeing, Figure 2 includes the following calls to action which optimize opportunities to prevent SRC incidence and related sequelae:

- Deliberating soccer specific head and body contact rule changes which have potential to mitigate undesirable head impacts;
- Implementing head-neck training grounded in sound clinical reasoning, which can be integrated into regular team training and devoid of foreseeable harm. For example, adopting the well-established FIFA-11 injury prevention model to integrate head-neck-SRC objectives, alongside feasibility and longitudinal impact evaluation.
- Investing in soccer-specific primary research to substantiate any significant effects of interventions alone (e.g., rule change vs. head-neck training interventions) or multimodal combinations. Examples could include randomized controlled trials directly comparing different head-neck training protocols or rule modifications.
- Recognizing bias limitations of research such as gender, population and varied performance levels when extrapolating clinically meaningful interpretations. Future research could further explore subgroup differences in more detail and expand on sex and gender considerations.
- Updating this review to discover a larger number of high-quality studies alongside evolving developments in this field of evidence.
- Increasing consistency in SRC outcome measures to facilitate pooled intelligence across studies and over time. A specific example could be adopting standardized metrics such as SRC incidence per 1000 player-hours across all future studies.

## 5. Conclusions

Interventions used in the prevention of SRC in adult soccer players were discovered in two identifiable domains of rule changes; and head-neck training interventions. Whilst these interventions were grounded in sound and well-reasoned mechanisms to mitigate SRC risks, evidence is preliminary, heterogeneous and insufficient to inform definitive practice recommendations. Sparce evidence represents research to reduce SRC in adult soccer players, and this is limited to investigating rule change and head-neck training and interventions. A larger volume of primary research is needed to determine clinically meaningful impacts of these and other conceivable interventions.

## Figures and Tables

**Table 1:** Inclusion and exclusion criteria. This table summarizes the inclusion and exclusion criteria for this review structured according to the PICO framework (Population, Intervention, Comparator, Outcomes). Abbreviations: sport-related concussion (SRC), Federation Internationale de Football Association (FIFA).

	Inclusion Criteria	Exclusion Criteria	Definition
Population	Football (soccer) players: adults (≥18 years), any sex or gender, any playing level (recreational, semi-elite, professional).	Any sport other than soccer; football codes other than soccer (e.g., American Football, Futsal, Australian Rules Football, Gaelic Football)	This review adopts the Federation Internationale de Football Association (FIFA) definition of soccer, which is the 11-player team sport of Association Football.
Intervention	Concussion prevention interventions including but not limited to intrinsic (neck strengthening exercises, neuromuscular training programs) and extrinsic factors (policy/rule changes, equipment).		SRC prevention interventions intending to prevent a SRC incident, including interventions designed to mitigate SRC risk factors [30].
Comparator	Usual care/standard care; Control group (those not exposed to preventative intervention).		Usual care resembles care and injury intervention activities normally provided in everyday practice [26] of soccer participation and SRC risk mitigation.
Outcomes	Sport-related concussion (SRC). SRC could be reported as medically diagnosed instances; athlete-reported instances; incidence rates.	Neurological signs, symptoms or diseases not diagnosed as SRC. Incidents of head impact without diagnosis of SRC.	Sport-related concussion (SRC) is defined as a traumatic brain injury due to direct head, neck or body impact, resulting in transmission of impulsive force to the brain that occurs in sports and exercise-related activities [1].
Study design	Randomized and non-randomized controlled trials Quasi randomized controlled trials Observational studies	Commentaries Individual case reports Qualitative studies Systematic reviews	
Publication type	Peer reviewed journals Published in English Full text, human-research articles		

**Table 2:** Included studies characteristics. This table provides a summary of included study characteristics, detailing study design, participants, competition level, intervention type, comparison timeline, and sport-related concussion (SRC) outcomes. Abbreviations: incidence rate (IR); not applicable (NA); National Collegiate Athlete Association (NCAA); not reported (NR); standard deviation (SD).

Study	Study Design; Surveillance Period	Participants (*N* = Number of Teams)	Level of Competition; Country	Intervention Type; Season of Implementation	Pre-Intervention Timeline	Sport-Related Concussion (SRC) Outcomes Measured
Beaudouin et al. [36]	Retrospective cohort study; 13 seasons 2000/01–2012/13	*N* = 18 teams Participants: NR, Male (100%) Age: NR	Professional—First German Bundesliga; Germany	Rule change: law of the game altered so that direct and deliberate ‘elbows to head’ were punished with a red card ^A^; 2007/08–2012/13 seasons	Prior to rule change; seasons 2000/01–2005/06	Concussion incidents, concussion incident rate (IR) per 1000 match hours Effect estimate: Incidence rate ratio
Beaudouin et al. [37]	Retrospective & prospective cohort study; 11 seasons 2006/07–2016/17	*N* = 18 teams Participants: NR, Male (100%) Age: NR	Professional—First German Bundesliga; Germany	Rule change: law of the game altered so that direct and deliberate ‘elbows to head’ were punished with a red card ^A^; 2006/07–2016/17 seasons	Each season prior (time trend)	Concussion incident rate (IR) per 1000 match hours
Bjørneboe et al. [38]	Prospective cohort study; 2 seasons 2010–2011	*N* = 12 teams (Control) *N* = 14 teams (Intervention) Participants: NR, Male (100%) Age: NR	Professional—Norwegian league Tippeligaen; Norway	Rule change: stricter rule enforcement, issuing automatic red cards for tackles with excessive force or intentional high elbows ^B^; 2011 season	Prior to rule enforcement; season 2010	Concussion incidents, acute injury incidents ^C^, acute injury IR ^C^ per 1000 match hours, head incident ^D^ rate per 1000 match hours Effect estimate: Acute injury IR ratio, head incident rate ratio
Klein et al. [4]	Prospective single- arm cohort clinical trial; 8 seasons 2014/15–2021/22	*N* = NA Participants = 162 Female 73 (45%), Male 89 (55%) Age = Mean 20.1	College—NCAA Division I & II; United States of America	Head-Neck Training: Cervical strengthening program utilizing NeckX device performed 3× per week for 12 weeks; 2021/22 season	Pre-intervention historical control; 2014/15–2020/21 seasons	Concussion incident rate (IR) ^E^ per athletic event ^F^
Reneker et al. [39]	Single-arm interventional study; 2 seasons 2017–2018	*N* = NA Participants = 75, Female 37 (49%), Male 38 (51%) Age = Mean 20.2 (SD = 1.46)	College—NCAA Division II; United States of America	Head-Neck Training: Multi-modal sensorimotor training delivered over 4 weeks (8 × face-to-face sessions & home exercise program); 2018 season	Pre-intervention historical control; 2017 season	Injury incident rate (IR) ^G^ per 1000 athlete exposures ^H^

^A^ In 2006, the International Football Association Board (IFAB) altered the law of the game so that direct and deliberate ‘elbows to head’ were punished with a red card. ^B^ Prior to the start of the 2011 season, the Football Association of Norway (NFF), the Norwegian Professional League Association (NTF), Oslo Sports Trauma Research Centre (OSTRC) and FIFA-Medical Assessment and Research Centre (F-MARC) agreed to stricter rule enforcement, issuing automatic red cards for tackles with excessive force or intentional high elbows. ^C^ Acute injury incidence data includes all reported injuries, not only concussion injuries. ^D^ A head incident occurred if contact to a player’s head caused play to be stopped by referee, and the injured player stayed down for over 15 s in pain or required medical treatment. ^E^ Concussion incidence rate included data from participating athletes from multiple sports, not just football athletes. ^F^ Athletic event defined as a game/match. ^G^ Injury incidence included concussions and traumatic lower extremity musculoskeletal injuries. ^H^ Athlete exposure was defined as one athlete participating in one-NCAA-sanctioned practice or competition.

**Table 3:** Summary of findings. Grouped by intervention category and population context, this table contains a summary of findings representing sports related concussion (SRC) according to absolute number of reported SRC (*n*SRC), SRC incident rates per 1000 match hours exposure (IR-SRC), and other relevant reporting characteristics. Abbreviations: absolute number of reported SRC (*n*SRC), SRC incident rates per 1000 match hours exposure (IR-SRC), incidence rate (IR); incidence rate ratio (IRR); *p* value was set at 0.05 significance for all studies.

Study	Level of Competition; Reported Sex	Intervention Provided	Pre-Intervention IR-SRC	Post-Intervention IR-SRC	Effect Estimate Concussion IRR	Key SRC Findings	Other SRC Findings	Effect Direction and Interpretation	Total Quality Assessment Score (Out of 27); Category
Rule change interventions									
Beaudouin et al. [36]	Professional 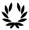 Males 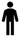	Rule change: law of the game altered so that direct and deliberate ‘elbows to head’ punished with a red card	IR-SRC: 0.67/1000 match hours (95% CI 0.51, 0.88)	IR-SRC: 0.48/1000 match hours (95% CI 0.34, 0.66)	Concussion IRR: 0.71 (95% CI 0.46, 1.09), *p* value 0.12	*n*SRC reduced by 29% following rule change but was not statistically significant (*p* = 0.12)	Pre-intervention: *n*SRC = 50 Post-intervention: *n*SRC = 35	No effect 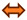 Elbow + head = red card 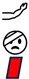	17 Fair 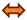
Beaudouin et al. [37]	Professional 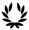 Males 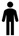	Rule change: law of the game altered so that direct and deliberate ‘elbows to head’ were punished with a red card	IR-SRC: 0.67/1000 match hours (95% CI 0.51, 0.88) 2000–2006 data from precursor study Beaudouin et al. (2017)	IR-SRC 0.53/1000 match hours (95% CI 0.42, 0.67) total seasons from 2006 to 2016)	Nil reported	Concussion IR increased year on year by 6.4% following the rule change. Total concussion IR for 11-season period remained lower compared to the rates reported from the 2000–2006 seasons in the precursor study (Beaudouin et al., 2017)	IR-SRC each season post-intervention: 2006/07: 0.41 (95% CI 0.17, 0.99) 2007/08: 0.48 (95% CI 0.22, 1.07) 2008/09: 0.32 (95% CI 0.12, 0.86) 2009/10: 0.41 (95% CI 0.17, 0.98) 2010/11: 0.58 (95% CI 0.28, 1.22) 2011/12: 0.49 (95% CI 0.22, 1.10) 2012/13: 0.57 (95% CI 0.27, 1.19) 2013/14: 0.58 (95% CI 0.28, 1.22) 2014/15: 0.49 (95% CI 0.22, 1.10) 2015/16: 0.81 (95% CI 0.44, 1.51) 2016/17: 0.74 (95% CI 0.39, 1.42)	No effect 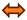 Elbow + head = red card 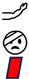	13 Poor 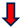
Bjørneboe et al. [38]	Professional 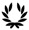 Males 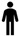	Rule change: stricter rule enforcement, issuing automatic red cards for tackles with excessive force or intentional high elbows	Acute injury IR ^A^: 16.9/1000 match hours (95% CI 13.6, 20.3)	Acute injury IR ^A^: 14.9/1000 match hours (95% CI 12.0, 17.8)	Acute injury IRR: 0.88 (95% CI 0.67, 1.16)	Stricter rule enforcement was associated with a lower head incident rate. No statistically significant difference was found in the overall acute injury IR or concussion frequency between the two groups.	Pre-intervention: *n*SRC = 3 Head incident ^B^ rate: 28.5 (95% CI 24.8, 32.3) Post-intervention: *n*SRC = 4 Head incident ^B^ rate: 23.2 (95% CI 19.9, 26.6) Head incident ^B^ rate ratio: 0.81 (95% CI 0.67, 0.99)	No effect 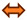 Strict tackle = red card 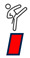	14 Fair 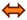
Training strategy interventions									
Klein et al. [4]	College 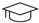 Males and females 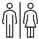	Cervical strengthening program utilizing NeckX device	Concussion IR ^C^ per athletic event 2020/21: 11.54	Concussion IR ^C^ per athletic event 2021/22: 6.67	Nil reported	Following implementation of the neck exercise protocol, concussion IR during the 2021/22 season was the lowest recorded value in the past eight athletic seasons at the University across all sports (incl. football)	Pre-intervention: Concussion IR ^C^ per athletic event 2014/15: 9.4 2015/16: 22.43 2016/17: 11.71 2017/18: 12.26 2018/19: 7.47 2019/20: 8.6	No effect 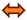 Neck strength program 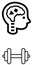	15 Fair 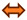
Reneker et al. [39]	College 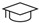 Males and females 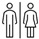	Multi-modal sensorimotor training program	Injury IR ^D^: 11.8/1000 athlete exposures	Injury IR ^D^: 8.94/1000 athlete exposures	Nil reported	A 27% reduction in injury rates (concussion and lower extremity combined) was observed following implementation of the sensorimotor training program but did not reach statistical significance (*p* = 0.18)	Not applicable	No effect 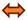 Neck+ sensor-motor program 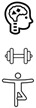	18 Fair 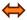

^A^ Data includes all acute injuries, not just concussion. ^B^ A head incident occurred if contact to a player’s head caused play to be stopped by referee, and the injured player stayed down for over 15 s in pain or required medical treatment. ^C^ Concussion incidence rate included data from participating athletes from multiple sports, not just football athletes. ^D^ Injury incidence included concussions and traumatic lower extremity musculoskeletal injuries.

## Data Availability

The original contributions presented in this study are included in the article/Supplementary Material. Further inquiries can be directed to the corresponding author.

## Supplementary Materials

The following supporting information can be downloaded at: https://www.mdpi.com/article/10.3390/healthcare14091200/s1, Supplementary File SA: PRISMA 2020 Checklist; Supplementary File SB: Search Strategy, B.1. MEDLINE, B.2. CINAHL, B.3. Embase, B.4. SPORTDiscus, B.5. PsycINFO; Supplementary File SC: The Synthesis Without Meta-analysis (SWiM) Checklist; Supplementary File SD: Quality Assessment Modified Downs and Black Checklist (Full).

## Abbreviations

The following abbreviations are used in this manuscript:

CI	confidence interval
CISG	Concussion in Sport Group
FIFA	Federation Internationale de Football Association
F-MARC	FIFA-Medical Assessment and Research Centre
GRADE	grading of recommendations, assessment, development and evaluations
IFAB	International Football Association Board
IR	incidence rate
IRR	incidence rate ratio
IR-SRC	SRC incident rates per 1000 match hours exposure
mTBI	mild traumatic brain injury
NACAA	National Collegiate Athlete Association
NA	not applicable
NFF	Football Association of Norway
NR	not reported
NRS	non-randomized studies
NTF	Norwegian Professional League Association
OSTRC	Oslo Sports Trauma Research Centre
PICO	Population, Intervention, Comparator, Outcomes
PRISMA	Preferred Reporting Items for Systematic Reviews and Meta-Analyses
RCT	Randomized controlled trial
RoB	Risk of bias
RSCI	repetitive sub concussive impacts
SD	standard deviation
SR	Systematic review
SRC	sport-related concussion
*n*SRC	absolute number of reported SRC
SWiM	Synthesis Without Meta-Analysis

## References

[1] Patricios J.S., Schneider K.J., Dvorak J., Ahmed O.H., Blauwet C., Cantu R.C., Davis G.A., Echemendia R.J., Makdissi M., McNamee M. (2023). Consensus statement on concussion in sport: The 6th International Conference on Concussion in Sport-Amsterdam, October 2022. Br. J. Sports Med..

[2] Harmon K.G., Drezner J.A., Gammons M., Guskiewicz K.M., Halstead M., Herring S.A., Kutcher J.S., Pana A., Putukian M., Roberts W.O. (2013). American Medical Society for Sports Medicine position statement: Concussion in sport. Br. J. Sports Med..

[3] McRae B., Stay S. (2024). Assessment and management of sport-related concussion in general practice. Aust. J. Gen. Pract..

[4] Klein J., Koch I., Delgadillo B.E., Chickness J., Blank J., Amos A., Tay K., Kelly E.A., Webber K., Benzinger B. (2024). Concussion Reduction in Division I and II Athletes: Effects of Simple Cervical Spine Exercise Regimen. Cureus.

[5] Yengo-Kahn A.M., Kelly P.D., Liles D.C., McKeithan L.J., Grisham C.J., Khan M.S., Lee T., Kuhn A.W., Bonfield C.M., Zuckerman S.L. (2020). The cost of a single concussion in American high school football: A retrospective cohort study. Concussion.

[6] Cunningham J., Broglio S.P., O’Grady M., Wilson F. (2020). History of Sport-Related Concussion and Long-Term Clinical Cognitive Health Outcomes in Retired Athletes: A Systematic Review. J. Athl. Train..

[7] AIS (2023). Concussion and Brain Health Position Statement 2023.

[8] AIS Australian Concussion Guidelines for Youth and Community Sport. https://sma.org.au/wp-content/uploads/2024/01/37382_Concussion-Guidelines-for-community-and-youth-FA-acc.pdf.

[9] FIFA FIFA Concussion Campaign—Suspect and Protect. https://inside.fifa.com/campaigns/concussion.

[10] Silverberg N.D., Iverson G.L., Cogan A., Dams O.C.K., Delmonico R., Graf M.J.P., Iaccarino M.A., Kajankova M., Kamins J., McCulloch K.L. (2023). The American Congress of Rehabilitation Medicine Diagnostic Criteria for Mild Traumatic Brain Injury. Arch. Phys. Med. Rehabil..

[11] Shibukawa K., Hoshikawa Y. (2024). Decrease in aerial challenges after revision of goal kick rules in Japan Professional Soccer League: Explorative study of the possibility of a risk reduction for head injury, concussion, and brain damage by a rule revision. Sci. Med. Footb..

[12] Library of Cogress (2026). Sports Industry: A Research Guide.

[13] Indharty R.S., Siahaan A.M.P., Rosarina, Susanto M., Tandean S., Risfandi M. (2023). Prevention of sports-related concussion in soccer: A comprehensive review of the literature. Ann. Med. Surg..

[14] Peek K., Duffield R., Cairns R., Jones M., Meyer T., McCall A., Oxenham V. (2023). Where are We Headed? Evidence to Inform Future Football Heading Guidelines. Sports Med..

[15] Putukian M., Echemendia R.J., Chiampas G., Dvorak J., Mandelbaum B., Lemak L.J., Kirkendall D. (2019). Head Injury in Soccer: From Science to the Field; summary of the head injury summit held in April 2017 in New York City, New York. Br. J. Sports Med..

[16] Al Attar W.S.A., Majrashi A., Bizzini M. (2025). Effectiveness of FIFA 11+ Injury Prevention Programs in Reducing Head and Neck Injuries, Including Concussion, Among Soccer Players: A Systematic Review and Meta-Analysis. Pediatr. Exerc. Sci..

[17] Shill I.J., Shepherd H.A., Eliason P.H., Kolstad A.T., Heyward O., Martens G., Peek K., Soligon C.A., King M.G., West S.W. (2025). Prevention strategies and modifiable risk factors for concussion: A systematic review and meta-analysis for the Female, woman and girl/or Athlete Injury pRevention (FAIR) consensus. Br. J. Sports Med..

[18] Harrison J., Lazzara B., Brown A., Esopenko C., Tosto Mancuso J., Levine J., Badillo B., Kaminski T., Peek K., Caccese J. (2025). The effectiveness of risk mitigation strategies for reducing the burden of heading in football: A systematic review. PROSPERO.

[19] Huber C.M., Patton D.A., Rownd K.R., Patterson Gentile C., Master C.L., Arbogast K.B. (2023). Neurophysiological Effects of Repeated Soccer Heading in Youth. J. Biomech. Eng..

[20] Basinas I., McElvenny D.M., Brooker F., Robertson S., van Hoecke Y., Kemp S., Pearce N., Gallo V., Cherrie J.W. (2023). Exposure assessment for repeated sub-concussive head impacts in soccer: The HEalth and Ageing Data IN the Game of football (HEADING) study. Int. J. Hyg. Environ. Health.

[21] Page M.J., McKenzie J.E., Bossuyt P.M., Boutron I., Hoffmann T.C., Mulrow C.D., Shamseer L., Tetzlaff J.M., Akl E.A., Brennan S.E. (2021). The PRISMA 2020 statement: An updated guideline for reporting systematic reviews. Syst. Rev..

[22] Vella T., Norton J., Foong K., Barbara M., Jones J., Moore S. (2025). A Systematic Review: Effectiveness of Interventions in Preventing Sport-Related Concussion in Adult Football (Soccer) Players. PROSPERO.

[23] Khalil H., Johns-Hayden A., Kynoch K. (2026). Guidance to including gray literature in systematic reviews-recommendations from an epidemiological study. J. Clin. Epidemiol..

[24] Aubry M., Cantu R., Dvorak J., Graf-Baumann T., Johnston K., Kelly J., Lovell M., McCrory P., Meeuwisse W., Schamasch P. (2002). Summary and agreement statement of the first international conference on concussion in sport, vienna 2001. Physician Sportsmed..

[25] Moore S., Musgrave C., Sandler J., Bradley B., Jones J.R.A. (2024). Early intervention treatment in the first 2 weeks following concussion in adults: A systematic review of randomised controlled trials. Phys. Ther. Sport.

[26] Turner K.M., Huntley A., Yardley T., Dawson S., Dawson S. (2024). Defining usual care comparators when designing pragmatic trials of complex health interventions: A methodology review. Trials.

[27] Fernainy P., Cohen A.A., Murray E., Losina E., Lamontagne F., Sourial N. (2024). Rethinking the pros and cons of randomized controlled trials and observational studies in the era of big data and advanced methods: A panel discussion. BMC Proc..

[28] Saldanha I.J., Adam G.P., Bañez L.L., Bass E.B., Berliner E., Devine B., Hammarlund N., Jain A., Norris S.L., Skelly A.C. (2022). Inclusion of nonrandomized studies of interventions in systematic reviews of interventions: Updated guidance from the Agency for Health Care Research and Quality Effective Health Care program. J. Clin. Epidemiol..

[29] Sarri G., Patorno E., Yuan H., Guo J.J., Bennett D., Wen X., Zullo A.R., Largent J., Panaccio M., Gokhale M. (2022). Framework for the synthesis of non-randomised studies and randomised controlled trials: A guidance on conducting a systematic review and meta-analysis for healthcare decision making. BMJ Evid. Based Med..

[30] Holm-Jensen A., Vlachos E., Storm L.K., Myburgh C. (2025). The Consistency of Primary, Secondary and Tertiary Prevention Definitions in the Context of Musculoskeletal Sports Injuries: A Rapid Review and Critical Exploration of Common Terms of Usage. Sports Med. Open.

[31] *Covidence Systematic Review Software*, Veritas, Health Innovation, Melbourne, Australia. http://www.covidence.org.

[32] Campbell M., McKenzie J.E., Sowden A., Katikireddi S.V., Brennan S.E., Ellis S., Hartmann-Boyce J., Ryan R., Shepperd S., Thomas J. (2020). Synthesis without meta-analysis (SWiM) in systematic reviews: Reporting guideline. BMJ.

[33] Greenhalgh T., Thorne S., Malterud K. (2018). Time to challenge the spurious hierarchy of systematic over narrative reviews?. Eur. J. Clin. Investig..

[34] Downs S.H., Black N. (1998). The feasibility of creating a checklist for the assessment of the methodological quality both of randomised and non-randomised studies of health care interventions. J. Epidemiol. Community Health.

[35] McCann R., Ramirez V., Schussler E., Martinez J. (2022). The Effect of Concussion History on Lower Extremity Injury Risk in College Athletes: A Systematic Review and Meta-Analysis. Int. J. Sports Phys. Ther..

[36] Beaudouin F., Aus der Fünten K., Tröß T., Reinsberger C., Meyer T. (2019). Head injuries in professional male football (soccer) over 13 years: 29% lower incidence rates after a rule change (red card). Br. J. Sports Med..

[37] Beaudouin F., der Fünten K.A., Tröß T., Reinsberger C., Meyer T. (2019). Time Trends of Head Injuries Over Multiple Seasons in Professional Male Football (Soccer). Sports Med. Int. Open.

[38] Bjørneboe J., Bahr R., Dvorak J., Andersen T.E. (2013). Lower incidence of arm-to-head contact incidents with stricter interpretation of the Laws of the Game in Norwegian male professional football. Br. J. Sports Med..

[39] Reneker J.C., Babl R., Pannell W.C., Adah F., Flowers M.M., Curbow-Wilcox K., Lirette S. (2019). Sensorimotor training for injury prevention in collegiate soccer players: An experimental study. Phys. Ther. Sport..

[40] Moreno A. Analysis of the German Football League (Bundesliga). https://towardsdatascience.com/analysis-of-the-german-football-league-bundesliga-35b8ee28765d/.

[41] O’Connor S.R., Tully M.A., Ryan B., Bradley J.M., Baxter G.D., McDonough S.M. (2015). Failure of a numerical quality assessment scale to identify potential risk of bias in a systematic review: A comparison study. BMC Res. Notes.

[42] McKee A.C., Mez J., Abdolmohammadi B., Butler M., Huber B.R., Uretsky M., Babcock K., Cherry J.D., Alvarez V.E., Martin B. (2023). Neuropathologic and Clinical Findings in Young Contact Sport Athletes Exposed to Repetitive Head Impacts. JAMA Neurol..

[43] Chen Y.L., Chou T.Y., Sung M.C., Huang Y.L. (2025). Sport-related Concussion Can be Prevented by Injury Prevention Program: A Systematic Review and Meta-analysis of Prospective, Controlled Studies. Sports Med. Open.

[44] Rennie G., Chesson L., Weaving D., Jones B. (2025). The effects of rule changes in football-code team sports: A systematic review. Sci. Med. Footb..

[45] Bizzini M., Dvorak J. (2015). FIFA 11+: An effective programme to prevent football injuries in various player groups worldwide-a narrative review. Br. J. Sports Med..

[46] Emery C.A., Meeuwisse W.H. (2010). The effectiveness of a neuromuscular prevention strategy to reduce injuries in youth soccer: A cluster-randomised controlled trial. Br. J. Sports Med..

[47] Eliason P.H., Galarneau J.M., Kolstad A.T., Pankow M.P., West S.W., Bailey S., Miutz L., Black A.M., Broglio S.P., Davis G.A. (2023). Prevention strategies and modifiable risk factors for sport-related concussions and head impacts: A systematic review and meta-analysis. Br. J. Sports Med..

[48] Lalji R., Snider H., Chow N., Howitt S. (2020). The 2015 U.S. Soccer Federation header ban and its effect on emergency room concussion rates in soccer players aged 10–13. J. Can. Chiropr. Assoc..

[49] Baker R., Bond B., Irwin G., Connelly S., Williams G. (2025). A systematic review of concussion education, knowledge, and attitudes in football. Sci. Med. Footb..

[50] Guilfoyle L., Kenny I.C., O’Sullivan K., Campbell M.J., Warrington G.D., Glynn L.G., Comyns T. (2024). Coaches of youth field sports as delivery agents of injury prevention programmes: How are we training the trainers? A scoping review. Br. J. Sports Med..

[51] Raihan N., Cogburn M. (2023). StatPearls [Internet]. https://www.ncbi.nlm.nih.gov/books/NBK538315/.

[52] Raftery M., Tucker R., Falvey É.C. (2020). Getting tough on concussion: How welfare-driven law change may improve player safety-a Rugby Union experience. Br. J. Sports Med..

[53] Perkins R.A., Bakhtiarydavijani A., Ivanoff A.E., Jones M., Hammi Y., Prabhu R.K. (2022). Assessment of brain injury biomechanics in soccer heading using finite element analysis. Brain Multiphys..

[54] Hallock H., Mantwill M., Vajkoczy P., Wolfarth B., Reinsberger C., Lampit A., Finke C. (2023). Sport-Related Concussion: A Cognitive Perspective. Neurol. Clin. Pract..

[55] Nowinski C.J., Rhim H.C., McKee A.C., Zafonte R.D., Dodick D.W., Cantu R.C., Daneshvar D.H. (2024). ‘Subconcussive’ is a dangerous misnomer: Hits of greater magnitude than concussive impacts may not cause symptoms. Br. J. Sports Med..

[56] Peek K., Andersen J., McKay M.J., Versteegh T., Gilchrist I.A., Meyer T., Gardner A. (2022). The Effect of the FIFA 11 + with Added Neck Exercises on Maximal Isometric Neck Strength and Peak Head Impact Magnitude During Heading: A Pilot Study. Sports Med..

[57] Attwood M.J., Roberts S.P., Trewartha G., England M.E., Stokes K.A. (2018). Efficacy of a movement control injury prevention programme in adult men’s community rugby union: A cluster randomised controlled trial. Br. J. Sports Med..

[58] Farley T., Barry E., Sylvester R., Medici A., Wilson M.G. (2022). Poor isometric neck extension strength as a risk factor for concussion in male professional Rugby Union players. Br. J. Sports Med..

[59] Morrissey S., Dumire R., Causer T., Colton A., Oberlander E., Frye D., Shepherd-Porada K., Frye L. (2019). The missing piece of the concussion discussion: Primary prevention of mild traumatic brain injury in student athletes. J. Emerg. Crit. Care Med..

[60] Fernández-Salido M., Alhambra-Borrás T., Casanova G., Garcés-Ferrer J. (2024). Value-Based Healthcare Delivery: A Scoping Review. Int. J. Environ. Res. Public Health.

[61] Corrick S., Lesyk N., Yang E., Campbell S., Villa-Roel C., Rowe B.H. (2023). Role of sex and gender in concussion outcome differences among patients presenting to the emergency department: A systematic review. Inj. Prev..

