# Supplementary material for: A Systematic Review of Peer-Reviewed Studies on Preventing Sport-Related Concussion (SRC) in Adult Football (Soccer): Mapping Sparce Evidence of Rule Changes and Head-Neck Training
Source: Healthcare (Basel). 2026 Apr 29;14(9):1200. doi: 10.3390/healthcare14091200 (PMC13163735; doi:10.3390/healthcare14091200)
Supplement: Supplementary file 1 [file healthcare-14-01200-s001.zip › HC_Supp D Quality Assessment Checklist.pdf]

Supplementary File D. Quality Assessment: Modified Downs and Black Checklist for each included study.

**Reporting**					
**Item**	Beaudouin et al[31]	Beaudouin et al[32]	Bjørneboe et al[33]	Klein et al[3]	Reneker et al[34]
**1 Is the hypothesis/aim/objective of the study clearly described?**	1 = Yes	1 = Yes	1 = Yes	1 = Yes	1 = Yes
**2 Are the main outcomes to be measured clearly described in the Introduction or Methods section?**	1 = Yes	1 = Yes	1 = Yes	1 = Yes	1 = Yes
**3 Are the characteristics of the patients included in the study clearly described?**	0 = No	0 = No	0 = No	1 = Yes	1 = Yes
**4 Are the interventions of interest clearly described?**	1 = Yes	1 = Yes	1 = Yes	1 = Yes	1 = Yes
**5 Are the distributions of principal confounders in each group of subjects to be compared clearly described?**	0 = No	0 = No	0 = No	0 = No	2 = Yes
**6 Are the main findings of the study clearly described?**	1 = Yes	1 = Yes	1 = Yes	1 = Yes	1 = Yes
**7 Does the study provide estimates of the random variability in the data for the main outcomes?**	1 = Yes	1 = Yes	1 = Yes	0 = No	0 = No
**8 Have all important adverse events that may be a consequence of the intervention been reported?**	0 = Unable to determine	0 = No	0 = Unable to determine	0 = No	0 = No
**9 Have the characteristics of patients lost to follow-up been described?**	0 = No	0 = No	0 = No	1 = Yes	1 = Yes
**10 Have actual probability values been reported (e.g. 0.035 rather than <0.05) for the main outcomes except where the probability value is less than 0.001?**	1 = Yes	1 = Yes	0 = No	1 = Yes	1 = Yes
**External Validity**					
**11 Were the subjects asked to participate in the study representative of the entire population from which they were recruited?**	1 = Yes	0 = Unable to determine	0 = Unable to determine	0 = Unable to determine	1 = Yes
**12 Were those subjects who were prepared to participate representative of the entire population from which they were recruited?**	1 = Yes	0 = Unable to determine	0 = No	0 = Unable to determine	1 = Yes

**13 Were the staff, places, and facilities where the patients were treated, representative of the treatment the majority of patients receive**?	1 = Yes	1 = Yes	1 = Yes	1 = Yes	0 = No
**Internal Validity**					
**14 Was an attempt made to blind study subjects to the intervention they have received?**	1 = Yes	0 = No	1 = Yes	0 = No	0 = No
**15 Was an attempt made to blind those measuring the main outcomes of the intervention?**	0 = No	0 = No	0 = No	1 = Yes	0 = No
**16 If any of the results of the study were based on "data dredging", was this made clear?**	1 = Yes	1 = Yes	1 = Yes	1 = Yes	1 = Yes
**17 In trials and cohort studies, do the analyses adjust for different lengths of follow-up of patients, or in case-control studies, is the time period between the intervention and outcome the same for cases and controls?**	1 = Yes	1 = Yes	1 = Yes	1 = Yes	1 = Yes
**18 Were the statistical tests used to assess the main outcomes appropriate?**	1 = Yes	1 = Yes	1 = Yes	1 = Yes	1 = Yes
**19 Was compliance with the intervention/s reliable?**	1 = Yes	1 = Yes	1 = Yes	0 = No	1 = Yes
**20 Were the main outcome measures used accurate (valid and reliable)?**	1 = Yes	1 = Yes	1 = Yes	1 = Yes	1 = Yes
**Internal Validity - Selection Bias**					
**21 Were the patients in different intervention groups (trials and cohort studies) or were the causes and controls (case-control studies) recruited from the same population?**	1 = Yes	1 = Yes	1 = Yes	1 = Yes	1 = Yes
**22 Were study subjects in different intervention groups (trials and cohort studies) or were the cases and controls (case-control studies) recruited over the same period of time?**	0 = No	0 = No	0 = No	1 = Yes	0 = No
**23 Were study subjects randomised to intervention groups?**	0 = No	0 = No	0 = No	0 = No	0 = No
**24 Was the randomised intervention assignment concealed from both patients and health care staff until recruitment was complete and irrevocable?**	0 = No	0 = No	0 = No	0 = No	0 = No

**25 Was there adequate adjustment for confounding in the analyses from which the main findings were drawn?**	0 = No	0 = No	0 = No	0 = No	0 = No
**26 Were losses of patients to follow-up taken into account?**	0 = No	0 = Unable to determine	0 = No	0 = No	1 = Yes
**Power**					
**27 Did the study have sufficient power to detect a clinically important effect where the probability value for a difference being due to chance is less than 5%?**	1 = Yes	0 = Unable to determine	1 = Yes	0 = No	0 = No
**Total Score**	17	13	17	15	18
