# Supplementary material for: A Systematic Review of Peer-Reviewed Studies on Preventing Sport-Related Concussion (SRC) in Adult Football (Soccer): Mapping Sparce Evidence of Rule Changes and Head-Neck Training
Source: Healthcare (Basel). 2026 Apr 29;14(9):1200. doi: 10.3390/healthcare14091200 (PMC13163735; doi:10.3390/healthcare14091200)
Supplement: Supplementary file 1 [file healthcare-14-01200-s001.zip › HC_Supp B Search Strategy.pdf]

Supplementary File B. Full search strategy. Search terms used in MEDLINE, CINAHL, Embase, SPORTDiscus and PsycINFO databases.

B1. MEDLINE
Date: 2024-09-06

Step #	Search string	Number of Records
1	Brain Concussion/	12446
2	concuss*.mp.	18805
3	mild traumatic brain injur*.mp.	7144
4	mtbi.mp.	4471
5	1 or 2 or 3 or 4	21439
6	Football.mp.	17566
7	soccer.mp.	16546
8	6 or 7	28225
9	Prevent*.mp.	2972067
10	(reduce or reduces or reduced or reduction* or reducing).mp.	4518245
11	Equipment.mp.	366975
12	(Mouthguard* or mouth guard* or mouthpiece* or mouth piece*).mp.	2694
13	(Helmet* or head gear* or headgear*).mp.	8234
14	((resistance or proprioception) adj2 (train* or exercis*)).mp.	25474
15	Neuromuscular training*.mp.	641
16	(risk or risks).mp.	3614533
17	((Policy or policies or rule* or training or game*) adj2 (chang* or amend* or modif* or regulation* or legislation*)).mp.	30049
18	((muscle* or neck or cervical or core) adj2 (strength* or training)).mp	60172
19	(rest or rests or resting or rested).mp.	334074
20	(Nutrition* or diet).mp.	956559
21	Vitamin*.mp.	319354
22	supplement*.mp.	469591
23	Education* adj2 (program* or intervention*)).mp.	84601
24	9 or 10 or 11 or 12 or 13 or 14 or 15 or 16 or 17 or 18 or 19 or 20 or 21 or 22 or 23	10537404
25	5 and 8 and 24	1550
26	limit 25 to (english language and yr="2001 -Current")	1483

B2. CINAHL
Date: 2024-09-06

Step #	Query	Limiters / Expanders	Results
S1	(MH "Brain Concussion")	Search modes - Proximity	6,301

S2	concuss*	Search modes - Proximity	8,412
S3	"mild traumatic brain injur*"	Search modes - Proximity	2,688
S4	Mtbi	Search modes - Proximity	1,476
S5	(S1 OR S2 OR S3 OR S4)	Search modes - Proximity	10,119
S6	Football	Search modes - Proximity	8,713
S7	Soccer	Search modes - Proximity	10,265
S8	S6 OR S7	Search modes - Proximity	15,754
S9	Prevent*	Search modes - Proximity	963,780
S10	(reduce or reduces or reduced or reduction* or reducing)	Search modes - Proximity	736,861
S11	Equipment	Search modes - Proximity	235,548
S12	(Mouthguard* or "mouth guard*" or mouthpiece* or "mouth piece*")	Search modes - Proximity	1,112
S13	(Helmet* or "head gear*" or headgear*)	Search modes - Proximity	2,819
S14	((resistance or propriocept*) N2 (train* or exercis*))	Search modes - Proximity	13,744
S15	"Neuromuscular training*"	Search modes - Proximity	398
S16	(risk OR risks)	Search modes - Proximity	1,194,937
S17	((Policy or policies or rule* or training or game*) N2 (chang* or amend* or modif* or regulation* or legislation*))	Search modes - Proximity	26,291
S18	((muscle* or neck or cervical or core) N2 (strength* or training))	Search modes - Proximity	44,854
S19	(rest or rests or resting or rested)	Search modes - Proximity	54,280
S20	(Nutrition* or diet)	Search modes - Proximity	307,979
S21	vitamin*	Search modes - Proximity	70,656
S22	supplement*	Search modes - Proximity	121,287
S23	((Education*) N2 (program* or intervention*))	Search modes - Proximity	57,845
S24	S9 OR S10 OR S11 OR S12 OR S13 OR S14 OR S15 OR S16 OR S17 OR S18 OR S19 OR S20 OR S21 OR S22 OR S23	Search modes - Proximity	2,770,923

S25	S5 AND S8 AND S24	Search modes - Proximity	766
S26	S5 AND S8 AND S24	**Limiters -** Publication Date: 20010101-20241231 Language: English Search modes - Proximity	741

B3. Embase
Date: 2024-09-06

Step #	Query	Results
1	Brain Concussion/	8962
2	concuss*.mp.	25101
3	mild traumatic brain injur*.mp.	10017
4	mtbi.mp.	7144
5	1 or 2 or 3 or 4	30717
6	Football.mp.	21710
7	soccer.mp.	16439
8	6 or 7	32629
9	Prevent*.mp.	3667074
10	(reduce or reduces or reduced or reduction* or reducing).mp.	6286627
11	Equipment.mp.	489772
12	(Mouthguard* or mouth guard* or mouthpiece* or mouth piece*).mp.	3924
13	(Helmet* or head gear* or headgear*).mp.	11944
14	((resistance or proprioception) adj2 (train* or exercis*)).mp.	38673
15	Neuromuscular training*.mp.	777
16	(risk or risks).mp.	5506368
17	((Policy or policies or rule* or training or game*) adj2 (chang* or amend* or modif* or regulation* or legislation*)).mp.	37641
18	((muscle* or neck or cervical or core) adj2 (strength* or training)).mp	126604
19	(rest or rests or resting or rested).mp.	505477
20	(Nutrition* or diet).mp.	1475330
21	Vitamin*.mp.	476241
22	supplement*.mp.	663516
23	Education* adj2 (program* or intervention*)).mp.	148881
24	9 or 10 or 11 or 12 or 13 or 14 or 15 or 16 or 17 or 18 or 19 or 20 or 21 or 22 or 23	14652579
25	5 and 8 and 24	2202
26	limit 25 to (english language and yr="2001 -Current")	2118

B4. SPORTDiscus
Date: 2024-09-06

Step #	Query	Limiters / Expanders	Results
S1	(MH "Brain Concussion")	Search modes - Proximity	4122
S2	concuss*	Search modes - Proximity	7110
S3	"mild traumatic brain injury" or mtbi	Search modes - Proximity	1156
S4	mtbi	Search modes - Proximity	503
S5	S1 OR S2 OR S3 OR S4	Search modes - Proximity	7526
S6	Football	Search modes - Proximity	192,098
S7	Soccer	Search modes - Proximity	68,344
S8	S6 OR S7	Search modes - Proximity	233, 328
S9	Prevent*	Search modes - Proximity	135,423
S10	(Reduce OR reduces OR reduced OR reduction* OR reducing)	Search modes - Proximity	133,013
S11	Equipment	Search modes - Proximity	88,135
S12	(Mouthguard* OR "mouth guard*" OR mouthpiece* OR "mouth piece*")	Search modes - Proximity	766
S13	(Helmet* OR "head gear*" OR headgear*)	Search modes - Proximity	6,423
S14	(Resistance OR proprioception) N2 (train* OR exercis*)	Search modes - Proximity	17,523
S15	Neuromuscular training*	Search modes - Proximity	1,570
S16	(Risk OR risks)	Search modes - Proximity	129, 336
S17	(Policy OR policies OR rule* OR training OR game*) N2 (chang* OR amend* OR modif* OR regulation* OR legislation*)	Search modes - Proximity	10,743
S18	(muscle* OR neck OR cervical OR core) N2 (strength* OR training)	Search modes - Proximity	34,592
S19	(Rest OR Rests OR resting OR rested)	Search modes - Proximity	32,643
S20	(Nutrition* OR diet)	Search modes - Proximity	138,079
S21	Vitamin*	Search modes - Proximity	14,346
S22	Supplement*	Search modes - Proximity	33,024

S23	(Education* N2 (Program* OR intervention*))	Search modes - Proximity	16,991
S24	(S9 OR S10 OR S11 OR S12 OR S13 OR S14 OR S15 OR S16 OR S17 OR S18 OR S19 OR S20 OR S21 OR S22 OR S23)	Search modes - Proximity	570,928
S25	S5 AND S8 AND S24	Search modes - Proximity	1,106
S26	S5 AND S8 AND S24	**Limiters -** Publication Date: 20010101-20241231; Language: English **Search modes -** Proximity	1,045

B5. Database: PsycINFO
Date: 2024-09-06

Step #	Search String	Number of Records
1	Brain Concussion/	16357
2	concuss*.mp.	25401
3	mild traumatic brain injur*.mp.	11256
4	mtbi.mp.	7258
5	1 or 2 or 3 or 4	29960
6	Football.mp.	23876
7	soccer.mp.	21744
8	6 or 7	37847
9	Prevent*.mp.	3306045
10	(reduce or reduces or reduced or reduction* or reducing).mp.	5126833
11	Equipment.mp.	386195
12	(Mouthguard* or mouth guard* or mouthpiece* or mouth piece*).mp.	2866
13	(Helmet* or head gear* or headgear*).mp.	9408
14	((resistance or proprioception) adj2 (train* or exercis*)).mp.	28020
15	Neuromuscular training*.mp.	705
16	(risk or risks).mp.	4222572
17	((Policy or policies or rule* or training or game*) adj2 (chang* or amend* or modif* or regulation* or legislation*)).mp.	46039
18	((muscle* or neck or cervical or core) adj2 (strength* or training)).mp	66912
19	(rest or rests or resting or rested).mp.	402806
20	(Nutrition* or diet).mp.	1037277
21	Vitamin*.mp.	331417
22	supplement*.mp.	529096
23	Education* adj2 (program* or intervention*)).mp.	171531

24	9 or 10 or 11 or 12 or 13 or 14 or 15 or 16 or 17 or 18 or 19 or 20 or 21 or 22 or 23	12039966
25	5 and 8 and 24	501
26	limit 25 to (english language and yr="2001 -Current")	328
